# Low all‐cause 90‐day mortality after revision knee arthroplasty: A study based on the Swedish Perioperative Register (SPOR), 2017–2022

**DOI:** 10.1002/jeo2.70682

**Published:** 2026-03-10

**Authors:** Sara Gavria, Jon Karlsson, Johanna Albert, Olof Sköldenberg, Jan Jakobsson

**Affiliations:** ^1^ Department of Clinical Sciences, Karolinska Institutet Danderyds Hospital Stockholm Sweden; ^2^ Department of Anaesthesia and Intensive Care Danderyds University Hospital Stockholm Sweden; ^3^ Department of Orthopaedics Sahlgrenska University Hospital, Sahlgrenska Academy Gothenburg Sweden; ^4^ Department of Orthopaedics Danderyds University Hospital Stockholm Sweden

**Keywords:** age, ASA‐class, knee arthroplasty, mortality, perioperative mortality rate (POMR), revision

## Abstract

**Purpose:**

Revision knee arthroplasty (rKA) is performed when a primary implant fails and is associated with a higher perioperative risk than primary arthroplasty. Early postoperative mortality after rKA in Sweden is poorly described. We aimed to estimate all‐cause mortality within 90 days after rKA in Sweden and to explore associations with patient characteristics and calendar year.

**Methods:**

Register‐based cohort study using data from the Swedish Perioperative Register (SPOR). All adults undergoing rKA (national classification of surgical procedures and the kod for knee revision atrhroplasty) during 2017–2022 were included (*n* = 2616). The primary outcome was all‐cause mortality within 90 days. Group comparisons used *χ* ^2^ tests and analysis of variance; multivariable logistic regression adjusted for calendar year, sex, age group, American Society of Anaesthesiologists (ASA) category and indication for revision.

**Results:**

Overall, 90‐day mortality was low (15/2616; 0.57%; 95% confidence interval [CI]: 0.34–0.92) with no significant variation by calendar year (*p* = 0.100). Thirty‐day mortality was 0.23% (6/2616; 95% CI: 0.10–0.47). In unadjusted analyses, age >80 years and ASA Class III/IV were associated with higher odds of 90‐day mortality; in adjusted analyses, age >80 years remained the only statistically significant predictor (adjusted odds ratio [OR] 13.63; 95% CI: 3.97–46.77). Sex, ASA class and indication were not statistically significant in the adjusted model. Age, ASA class and indication showed substantial interaction in relation to 90‐day mortality.

**Conclusions:**

Ninety‐day mortality after rKA in Sweden was low and remained stable between 2017 and 2022. Advanced age, higher ASA class and revision due to periprosthetic joint infection appeared to be the dominant risk factors. Preoperative preparation, medical optimization and, where feasible, prehabilitation of older and frail patients should be considered.

**Level of Evidence:**

Not applicable, quality register‐based study.

**Abbreviations**
ACS NSQIP: American College of Surgeons—National Surgical Quality Improvement Program
ASA: American Society of Anaesthesiologists Physical Status Classification
BMI: body mass index
CRP: C‐reactive protein
CRR: cumulative revision rate
ERAS: enhanced recovery after surgery
KA: knee arthroplasty
LIA: local infiltration analgesia
PJI: periprosthetic joint infection
POMR: perioperative mortality rate
pTKR: primary total knee replacement
rKA: revision knee arthroplasty
rKR: revision knee replacement
SKAR: Swedish Knee Arthroplasty Register
TKA: total knee arthroplasty

## INTRODUCTION

Total knee arthroplasty (TKA) is one of the most frequently performed orthopaedic procedures [1]. The Swedish Arthroplasty Register reports a 10‐year revision rate of approximately 5%–6% after primary implantation [14]. Perioperative mortality rate (POMR) is a widely used indicator of quality of care [10], and reports from US and UK registries describe mortality after revision knee arthroplasty (rKA). However, contemporary Swedish data on early mortality after rKA and its association with patient factors are limited.

The aim of this study was to estimate all‐cause mortality within 90 days after rKA in Sweden (2017–2022) using data from the Swedish Perioperative Register (SPOR). We also evaluated the association between patients' characteristics and mortality risk, and whether annual POMR changed during the study period.

We hypothesized that annual 90‐day mortality, after adjustment for patient characteristics, decreased over the study period.

## MATERIALS AND METHODS

### Study design

This register‐based cohort study used data from the SPOR. The design enabled linkage of patient characteristics, indication and perioperative time stamps for rKA performed between 1 January 2017 and 30 June 2022.

### Study population

rKA procedures were identified in SPOR using the national procedure code (national classification of surgical procedures and the kod for knee revision atrhroplasty). Inclusion criteria were age ≥18 years and a recorded revision procedure performed between 1 January 2017 and 30 June 2022. Records with invalid personal identifiers that prevented outcome assessment were excluded. For patients with multiple revisions, only the most recent procedure was included when assessing mortality within 90 days.

### Outcome

The primary outcome was all‐cause mortality within 90 days after rKA, defined in line with the Utstein consensus report [2]. Secondary outcomes were all‐cause mortality on the day of surgery (Day 0), within 7 days and within 30 days.

### Variables

Variables included age, sex, American Society of Anaesthesiologists (ASA) class, body mass index (BMI), indication for revision, duration of surgery (*skin‐to‐skin*), anaesthesia time, theatre occupancy time, recovery room stay, calendar year of procedure and mortality within 90 days.

### Categorization

Age was summarized descriptively in three categories: 18–65 years, 66–80 years and ≥81 years. Sex was recorded as female or male. ASA class was recorded as ASA I–IV and also grouped as ASA low (I–II) and ASA high (III–IV). Perioperative times (duration of surgery, anaesthesia, theatre occupancy and recovery room stay) were reported in minutes. Indications were grouped as mechanical complication, periprosthetic joint infection (PJI), knee osteoarthritis or miscellaneous. Calendar year was treated as a categorical variable (2017–2022).

#### Post hoc

For regression analyses, age was specified a priori as a binary covariate (18–80 vs. >80 years) to avoid sparse cells and improve model stability.

### Statistics

Continuous variables (e.g., age and perioperative times) were summarized as medians with 95% confidence intervals (95% CIs). The Shapiro–Wilk test indicated non‐normal distributions. Categorical variables, including sex, ASA class and mortality, were summarized as counts and percentages. Mortality rates were calculated as the number of deaths divided by the number of procedures. Continuous variables were compared using non‐parametric testing; Mann–Whitney *U* test and Kruskal–Wallis test. Categorical variables were compared using the *χ* ^2^ test when expected cell counts were sufficient; otherwise, Fisher's exact test was used. Binary logistic regression was used to estimate odds ratios (ORs) for all‐cause 90‐day mortality, both unadjusted and adjusted for sex, age, ASA class and indication for revision. POMRs are presented as percentages with 95% confidence intervals, acknowledging the low number of events. A *p* value < 0.05 was considered statistically significant. Data were managed in Microsoft Excel 365 and analysed using IBM SPSS Statistics version 29.0 (IBM Corp.).

## RESULTS

A total of 2964 rKA procedures were identified in SPOR; after accounting for repeat procedures (348 additional revisions), 2616 unique patients were included in the analytic cohort. Table 1 presents demographic and perioperative characteristics (*n* = 2616). The cohort comprised 55.7% females, with a median age of 70 range 19 and 96 years. ASA Class II was the most common category (59%). Baseline characteristics did not differ significantly across calendar years. The most common indication for revision was mechanical complication (52%), followed by PJI (19%), osteoarthritis (15%) and miscellaneous indications (14%). The annual distribution of indications varied over the study period; the proportion of PJI ranged from 17% in 2019 to 22% in 2021.

**Table 1:** Patients' characteristics and all‐cause 30‐ and 90‐day mortality for the study cohort.

		Missing information
Sex, male/female, *n*	1159/1456	1
Age, years, median and 95% CI	70 (70–71)	0
ASA class, *n*	252/1535/785/23	21
Indication, *n*		272
Mechanical complication	1229	
Periprosthetic joint infection	446	
Osteoarthritis	346	
Miscellaneous	323	
Year, *n*		0
2017	364	0
2018	474	0
2019	558	0
2020	432	0
2021	463	0
2022 (January–June)	325^a^	0
Mortality Day 30, *n*	6	0
Mortality Day 90, *n*	15	0

Abbreviations: ASA, American Society for Anesthesiologists functional class; CI, confidence interval; *n*, number of patients.

a Six‐month data only.

Table 2 summarizes perioperative observations. There were no significant differences in duration of surgery, anaesthesia, theatre occupancy time or recovery room stay across the study period. Completion of the World Health Organization (WHO) Safe Surgery Checklist was recorded in 82% of cases and increased from 79% in 2017 to 90% in 2022 (*p* = 0.001).

**Table 2:** Perioperative observations.

Theatre occupancy time min. median (95% CI)	224 (220–229)
Duration of anaesthesia min. median (95% CI)	183 (179–188)
Duration of surgery min. median (95% CI)	117 (114–122)
Recovery room stay min. median (95% CI)	195 (191–200)
Blood loss ml. median (95% CI)	300 (300–350)
WHO safe surgery checklist completed, *n* (%)	
Yes	2156 (82.0)
No	460 (18.0)

Abbreviations: CI, confidence interval; min., minutes; *n*, number of patients; WHO, World Health Organization.

**Figure 1:**
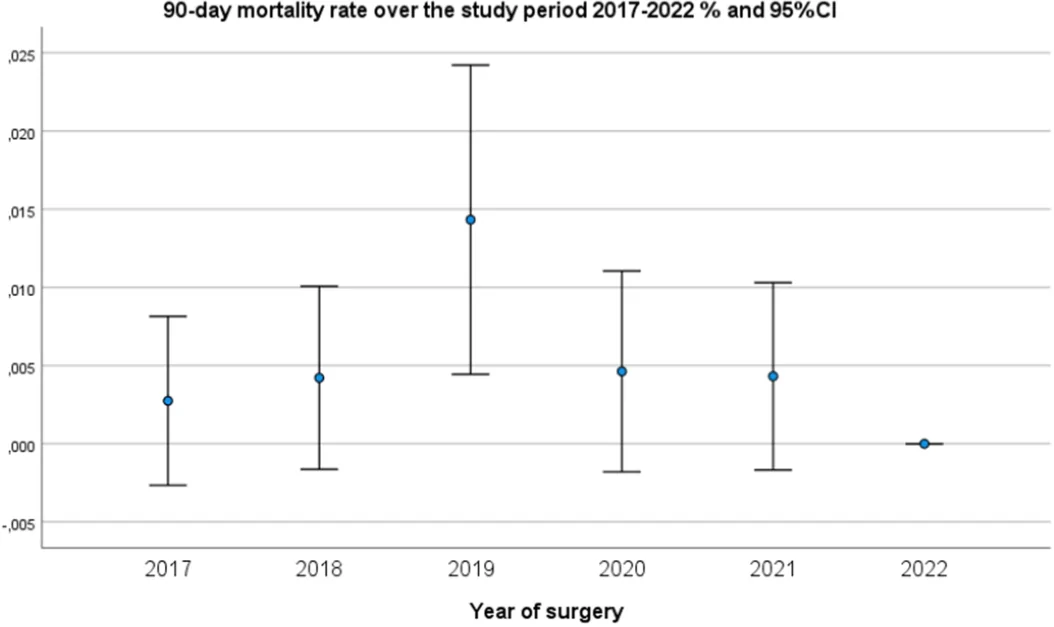
Percent and 95% confidence interval (CI) for the 90‐day mortality rate over the years.

Table 3 summarizes mortality outcomes. Ninety‐day mortality was 0.57% (*n* = 15) with no significant variation between years. Figure 1 shows the annual 90‐day mortality rate and corresponding 95% CIs. Thirty‐day mortality was 0.23% (*n* = 6) and did not differ significantly across years. No deaths occurred on the day of surgery, and two deaths occurred within the first week.

**Table 3:** All‐cause Day‐30 and Day‐90 mortality over the study period 2017–2022.

	All (*n* = 2616)	2017 (*n* = 364)	2018 (*n* = 474)	2019 (*n* = 558)	2020 (*n* = 432)	2021 (*n* = 463)	2022^a^ (*n* = 325)	*p* Value
30‐day mortality								0.2^b^
Alive, *n*	2610	364	473	554	431	463	325	
Deceased, *n* (%; 95% CI)	6 (0.23; 0.10–0.47)	0 (0)	1 (0.21; 0.02–0.98)	4 (0.72; 0.24–1.70)	1 (0.23; 0.02–1.08)	0 (0.0)	0 (0.0)	
90‐day mortality								0.1^b^
Alive, *n*	2601	363	472	550	430	461	325	
Deceased, *n* (%; 95% CI)	15 (0.57; 0.34–0.92)	1 (0.27; 0.03–1.28)	2 (0.42; 0.09–1.35)	8 (1.43, 0.68–2.69)	2 (0.46; 0.10–1.48)	2 (0.43; 0.09–1.38)	0 (0)	

*Note*: Presented in numbers and mortality rate (%; 95% CI).

Abbreviations: CI, confidence interval; *n*, number of patients.

^a^ Data from 2022 were collected across 6 months (January–June).

b Fisher's exact test.

**Figure 2:**
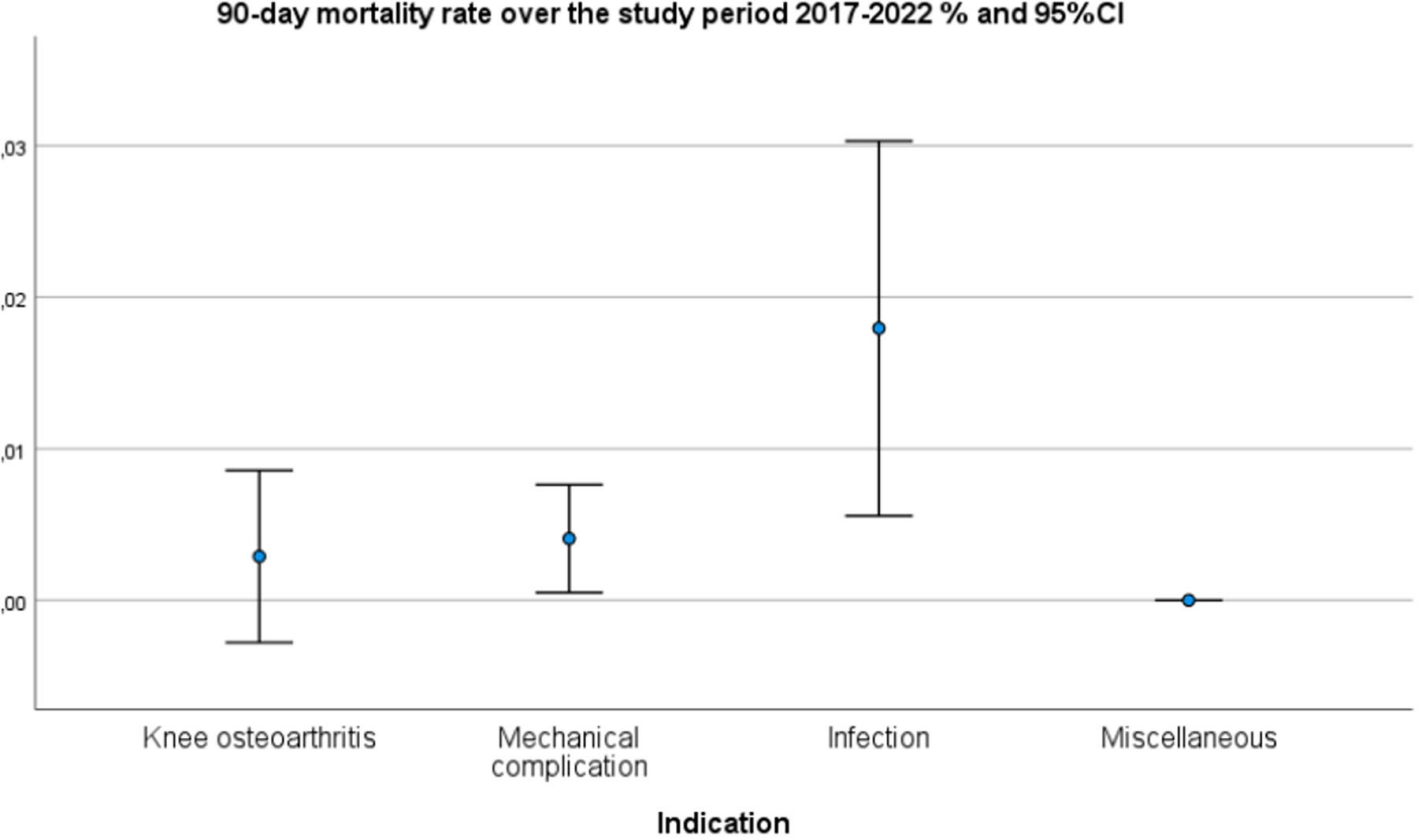
All cause 90‐day mortality rates and 95% CI in relation to indication for revision. CI, confidence interval.

Table 4 compares characteristics among patients alive and deceased at 90 days. Mortality did not differ by sex. Patients who died were on average 13 years older than survivors (*p* = 0.001). No deaths occurred among patients aged 18–65 years; mortality increased with age (*p* = 0.001). Mortality also increased with higher ASA class. Differences in mortality were observed by indication, with PJI associated with a 1.36% 90‐day mortality (*p* = 0.008; Figure 2).

**Table 4:** Patients' characteristics among patients alive and deceased at Day 90.

	Alive	Deceased	*p* Value
Sex female/male, *n* (row %)	1449/1151	7/8 (0.48/0.69)	n.s.
Age year median (95% CI)	70 (70–71)	83 (82–86)	0.001
18–65 years, *n*	842	0	
66–80 years	1455	4 (0.27)	
80+ years	304	11 (3.49)	
ASA,^a^ *n* (row %)			0.001
1	252	0	
2	1532	3 (0.20)	
3	776	9 (1.15)	
4	21	2 (8.70)	
ASA low I–II	1784	3 (0.17)	0.001
ASA high III–IV	797	11 (1.36)	
Indication, *n* (row %)			0.008
Mechanical complication	1224	5 (0.41)	
Infectious	438	8 (1.79)	
Osteoarthritis	345	1 (0.29)	
Miscellaneous	323	0	

Abbreviations: ASA, American Society for Anesthesiologists functional class; CI, confidence interval; *n*, number of patients; n.s., not significant.

^a^ Missing information about the ASA class for one deceased patient.

Table 5 presents unadjusted and adjusted logistic regression analyses for 90‐day mortality. Compared with 2017, no calendar year showed a statistically significant difference in mortality risk. No deaths were observed in 2022; data for 2022 cover January–June only. Age >80 years and ASA Class III–IV were strongly associated with mortality in unadjusted analyses. In the adjusted model, age >80 years remained the only statistically significant predictor. Sex and indication were not statistically significant. Revision due to infection showed elevated point estimates (crude OR 6.3; adjusted OR 4.1). The attenuation from crude to adjusted estimates suggests interaction between age, ASA class and indication in relation to 90‐day mortality.

**Table 5:** Binary logistic regression assessing OR with 95% CI for annual 90‐day mortality rate associated with revision knee arthroplasty unadjusted and adjusted for patients' characteristics, sex, age, ASA‐class and indication.

Variable	Category	Frequency	OR crude	95% CI crude	*p* Value crude	OR adjusted	95% CI adjusted	*p* Value adjusted
POMR	2017	364	Reference category	—	—	Reference category	—	—
	2018	474	1.54	0.14–17.03	0.73^a^	1.69	0.15–19.50	0.67^a^
	2019	558	5.28	0.66–42.39	0.12^a^	5.43	0.62–47.74	0.13^a^
	2020	432	1.69	0.15–18.69	0.67^a^	1.76	0.15–20.40	0.65^a^
	2021	463	1.58	0.14–17.44	0.71^a^	1.20	0.10–13.94	0.89^a^
	2022^b^	325	No events	—	Not estimable	No events	—	Not estimable
Sex	Female	1456	Reference category	—	—	Reference category	—	—
	Male	1159	1.44	0.52–3.98	0.48^a^	1.03	0.33–3.23	0.96^a^
Age group	18–80 years	2613	Reference category	—	—	Reference category	—	—
	>80 years	327	20.78	6.58–65.66	<0.001^a^	13.63	3.97–46.77	<0.001^a^
ASA category	ASA low (I–II)	1787	Reference category	—	—	Reference category	—	—
	ASA high (III–IV)	808	8.20	2.28–29.50	<0.001^a^	3.80	0.99–14.60	0.052^a^
Indication	Osteoarthritis	346	Reference category			Reference category		
	Mechanical complication	1229	1.41	0.16–12.10	0.75	1.23	0.14–11.04	0.85
	Infectious	446	6.30	0.78–50.62	0.08	4.1	0.47–35.83	0.20
	Miscellaneous	323	0		0.99	0		0.99

*Note*: Bold *p* value describes statistically significant.

Abbreviations: ASA, American Society of Anaesthesiologists; CI, confidence interval; OR, odds ratio; POMR, perioperative mortality rate.

^a^ Binary logistic regression.

^b^ Data from 2022 were collected across 6 months (January–June).

## DISCUSSION

The main findings were that 90‐day mortality after rKA was low (0.57%; 15 patients out of 2616), and 30‐day mortality was 0.23% (6 patients out of 2616). We found no evidence of a change in annual POMR over the study period. In the adjusted model, age >80 years was the only variable independently associated with 90‐day mortality.

These mortality estimates are consistent with previous reports from the United Kingdom and the United States. Using the UK National Joint Registry, Sabah et al. reported a 90‐day mortality of 0.7% among patients undergoing first revision TKA between 2009 and 2019 [11]. In the US American College of Surgeons—National Surgical Quality Improvement Program (ACS NSQIP), Sinclair et al. reported a 30‐day mortality of 0.36% after revision TKA between 2011 and 2019 [13]. No previous Swedish study has described early mortality after knee revision surgery. In contrast, Swedish data for primary KA show substantially lower early mortality [6], consistent with the Swedish Arthroplasty Register annual report [14]. Together, these comparisons underline that rKA is performed with high safety standards in Sweden, but carries a higher systemic risk profile than primary arthroplasty.

Siddiqi et al. analysed outcomes after hip and knee revision surgery in the US NSQIP database over 2008–2018 and reported a temporal reduction in 30‐day mortality [12]. We did not observe a similar trend, although the low number of events in the present cohort limits the ability to detect modest changes over time.

We observed no statistically significant difference in mortality between women and men. Male sex did not emerge as an independent predictor of death in adjusted analyses, consistent with the findings of Sinclair et al. [13]. Patient age distribution did not change significantly over the study period. No deaths occurred among patients younger than 65 years, whereas patients aged >80 years had a significantly higher mortality rate than those aged 66–80 years in both crude and adjusted analyses. This is in line with prior data demonstrating increasing mortality with advancing age after revision TKA [12]. Higher ASA class was associated with mortality in crude analyses. The impact of ASA class on early mortality has been demonstrated in Swedish studies of primary KA and revision hip arthroplasty during similar periods [3, 6], and ASA class is a well‐established predictor of complications and mortality across surgical specialties [4].

Ninety‐day mortality was higher among patients revised for infection. This pattern is consistent with Tyas et al., who reported PJI as an independent predictor of 90‐day mortality [15], and with large registry data indicating higher longer‐term mortality after septic revision compared with aseptic revision [8].

Advanced age and higher ASA class are expected risk factors. Although age is not a formal component of ASA classification, increasing age is associated with reduced physiological reserve and greater comorbidity burden. The observed attenuation of effect estimates after adjustment suggests that age, ASA class and indication are interrelated in their association with early mortality.

The present findings do not allow firm recommendations on risk mitigation, but they support a focus on preoperative optimization in older and frail patients. Where feasible, multimodal prehabilitation programmes combining exercise, nutritional and cognitive components may reduce complications [7, 9].

A key strength of this study is the use of SPOR, a validated national quality registry with broad coverage across Swedish hospitals, including both high‐ and low‐volume centres [5]. Several limitations should be considered. We did not assess surgical details (e.g., technique, prosthesis type or indication subcategories) or prior revision history. We included all revision procedures (partial and total, with or without patellar component). The registry does not contain information on antibiotic treatment for infection revisions, detailed comorbidity profiles beyond ASA class or cause of death, and we could not evaluate the clinical course leading to fatal outcomes.

## CONCLUSIONS

This nationwide study based on SPOR provides contemporary benchmark estimates of early mortality after rKA in Sweden. Ninety‐day mortality was 0.57% (15 patients out of 2616) and 30‐day mortality was 0.23% (6 patients out of 2616) between 2017 and 2022, with no evidence of temporal change. Mortality risk increased markedly in patients aged >80 years and was higher in patients with higher ASA class and in those revised for PJI. Continued efforts to optimize older and frail patients before surgery, including consideration of prehabilitation, appear warranted.

## AUTHOR CONTRIBUTIONS

*Concept and protocol*: Jan Jakobsson. *Data handling and analysis*: Sara Gawria and Jan Jakobsson (supported by Fredrik K. Johansson, KI statistician). *Writing manuscript*: Sara Gawria and Jan Jakobsson. *Manuscript review and input*: Johanna Albert, Olof Sköldenberg and Jon Karlsson. *Submission*: Jan Jakobsson.

## CONFLICT OF INTEREST STATEMENT

Jan Jakobsson is a paid safety physician for AstraZeneca, CTC and Mölnlycke. The remaining authors declare no conflict of interest.

## ETHICS STATEMENT

This study protocol was approved by the Ethical Review Board 2022‐02521‐02, Uppsala Department 2 of Medicine. This was a register‐based study using data from the national quality register (SPOR).

## Data Availability

Data are available on request from the National Quality Register SPOR.
